# Influence of Vanadium-Titanium Slag Substitution on Properties and Microstructure of Blast Furnace Slag-Steel Slag-Desulfurization Gypsum Gel System

**DOI:** 10.3390/gels12010003

**Published:** 2025-12-19

**Authors:** Junyao Liu, Siqi Zhang, Huifen Yang, Wen Ni, Dongshang Guan, Xingyang Xu, Yu Zhan

**Affiliations:** 1 School of Resources and Safety Engineering, University of Science and Technology Beijing, Beijing 100083, China; 15933769317@163.com (J.L.); niwen@ces.ustb.edu.cn (W.N.); 17613270209@163.com (D.G.); 18355781752@163.com (X.X.); m202310018@xs.ustb.edu.cn (Y.Z.); 2 Key Laboratory of Ministry of Education of China for Efficient Mining and Safety of Metal Mines, University of Science and Technology Beijing, Beijing 100083, China; 3 Institute of Mineral Resources, University of Science and Technology Beijing, Beijing 100083, China

**Keywords:** solid-waste gels materials, vanadium-titanium slag, compressive strength, C–S–H gels, ettringite

## Abstract

The comprehensive utilisation of solid waste is a primary approach to enhancing the utilisation efficiency of mineral resources. However, vanadium-titanium slag has long faced insufficient resource utilisation due to its low activity. To address this issue, this study integrated macro and micro analytical methods to systematically investigate the effect of mechanical grinding on the activity of vanadium-titanium slag, as well as its performance when partially replacing blast furnace slag in the system of slag—converter steel slag-desulfurization gypsum ternary gel system. Additionally, the hydration mechanism of this cementitious system was analysed. The research results indicate that mechanical grinding can significantly improve the activity index of vanadium-titanium slag and increase its specific surface area. Replacing an appropriate amount of slag with vanadium-titanium slag in the slag-steel slag-desulfurization gypsum ternary gel system can effectively enhance the mechanical properties of the cementitious system. The optimal mix proportion of vanadium-titanium slag:slag:steel slag:desulfurization gypsum as 10.5:31.5:42:16 with a water-to-binder ratio of 0.32, under which the 28-day compressive strength of the specimen reached 33.50 MPa. Through multiple microscopic analysis techniques, it was found that in the alkaline environment and sulfate excitation (provided by steel slag hydration and desulfurization gypsum), the cementitious system generates hydration products such as ettringite (AFt), C–S–H, and C–A–S–H gels. Some unreacted vanadium-titanium slag particles are wrapped and intertwined by hydrated calcium silicate (aluminium) gels, forming a stable dendritic structure that provides support for the system’s strength development.

## 1. Introduction

**Figure 1:**
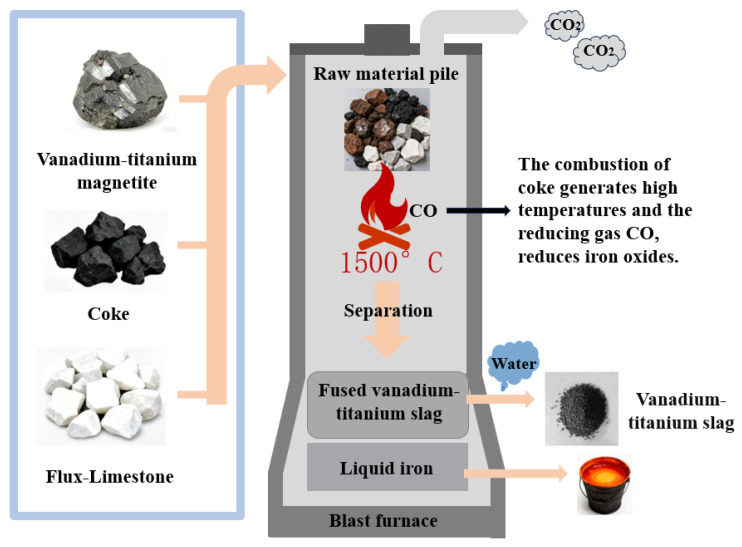
The generation process of vanadium-titanium slag.

Vanadium-titanium magnetite, a compound mineral resource characterised by its abundance of reserves, contains iron, vanadium, titanium and a variety of valuable metal elements. With regard to the global distribution of vanadium-titanium magnetite resources, China is third in global rank, with an estimated 18 billion tons of resources [1,2]. Vanadium-titanium slag, a metallurgical solid waste produced during the smelting of pig iron by vanadium-titanium magnetite, as illustrated in Figure 1, possesses an overall chemical composition that closely resembles that of ordinary slag, which is primarily composed of SiO_2_, CaO, Al_2_O_3_, and other elements [3]. However, due to the elevated TiO_2_ content in vanadium-titanium slag and the comparatively diminished content of CaO, the elevated degree of polymerization of silico-oxygen tetrahedron and the substantial “crystal-to-glass ratio” result in a diminished hydraulic activity (The ability of a material to form hydrated products with certain strength and stability after reacting with water.) relative to that of conventional slag [4,5]. Furthermore, an increase in the TiO_2_ content results in a reduction in activity and an enhancement of wearability. These characteristics render the utilisation of vanadium-titanium slag for effective resource management challenging. Presently, the annual production of vanadium-titanium slag in China amounts to tens of millions of tons. This material is currently only able to be stockpiled as solid waste, resulting in significant land occupation and associated environmental pollution and resource wastage [6].

In view of the above situation, extant domestic research on the utilisation of vanadium-titanium slag is chiefly concentrated on two areas. It is evident that the primary focus of utilisation in this context pertains to the recovery of titanium resources present within vanadium-titanium slag, with no consideration given to the recycling of titanium. The former are prioritised for the extraction of titanium, with methods including the acid method, fusion electrolysis and TiO_2_ enrichment. It is evident that these methods are frequently intricate processes, and they carry the risk of secondary pollution, which renders their application and promotion on a large scale challenging [7,8]. The latter process does not involve the extraction of titanium, the utilisation of vanadium, and the use of titanium slag as feedstock for specific materials, such as building materials, glass, adsorption and functional materials [9,10].

At present, extensive research foundations have been established domestically and internationally for the steel slag-slag-desulfurised gypsum cementitious system. In the conventional ground granulated blast furnace slag-steel slag-desulfurised gypsum cementitious system, the presence of steel slag and desulfurization gypsum has been observed to stimulate the activity of slag through the process of hydration reaction [11,12]. Ke et al. employed trace amounts of carbide slag (CS) to enhance the early-age performance of the steel slag–slag–desulfurization gypsum system. The results indicated that with a steel slag content of 5%, the addition of 0.6% CS significantly improved the system’s performance: the final setting times of three mix formulations were reduced from 17.2 to 19.1 h to less than 5.5 h, while the 3-day compressive strength increased by more than 16-fold. Mechanistically, CS elevated the system pH from 11.4 to 12.5, thereby promoting the dissolution of desulfurization gypsum and accelerating the formation of ettringite and C–(A)–S–H gels. Furthermore, it was observed that the enhancing effect of CS gradually diminished as the SS content increased [13]. Li et al. obtained the optimal mix ratio of slag:steel slag:desulfurised gypsum as 6:3:1 via the cross-experiment method, with the 28-day compressive strength reaching 40.40 MPa [14]. Yang et al. found through experiments that when the content of steel slag was fixed at 50% and the content of desulfurised gypsum was 10%, the cementitious material could achieve the optimal strength performance, and its 28-day strength reached 30.69 MPa. In addition, they illustrated that hydration products had already formed inside the test blocks in the early hydration stage of the slag-steel slag-gypsum cementitious material system [15].

In comparison with conventional slag, the activity of vanadium-titanium slag is reduced. Research on its application in cementitious materials is relatively limited [16]. Xue et al. utilised vanadium-titanium slag from Chengde Iron and Steel Company to conduct experimental research on the development of cementitious materials for mine filling. The results showed that the 28-day compressive strength of specimens activated by acidic activators did not exceed 1.0 MPa, and the effect of alkaline activators was even poorer; after further optimisation of activation experiments, the 28-day compressive strength reached approximately 3 MPa, which met the strength requirements for filling materials [17]. Gao et al. conducted an analysis of the effect of vanadium-titanium slag treated with different grinding times on cement activity. The study concluded that vanadium-titanium slag mainly exerts its activity at a later stage in the cement system, and that finer grinding helps to enhance its activity [18]. Shi et al. [19] found that vanadium-titanium slag had a significant effect on the consistency and setting time of cement, although the type of hydration products remained unaltered. Furthermore, the water rigidity of the slag was enhanced in high alkaline environments. As demonstrated by Wang et al., the incorporation of vanadium-titanium slag has been shown to enhance the degree of extension of cement and reduce the heat of hydration of the system through experimentation. This was achieved by utilising vanadium-titanium slag with varying degrees of fineness as a concrete admixture [20]. These findings suggest that vanadium-titanium slag has the potential to enhance or improve certain aspects of the properties of gels materials (Through the synergistic preparation from solid waste, it is a material capable of undergoing physical or chemical changes to solidify and harden from a slurry state, thereby firmly binding together granular or bulk materials such as sand.), which opens up the possibility of realising the above research ideas.

It is proposed to introduce vanadium-titanium slag into the slag-steel slag-desulfurised gypsum system after improving its activity via physical or chemical methods, where vanadium-titanium slag can partially or fully replace slag, thereby providing a low-cost and large-scale resource utilisation approach for vanadium-titanium slag. Therefore, the present study investigates the utilisation of various solid waste materials, including vanadium-titanium slag, slag, steel slag, desulfurization gypsum (from Shougang Shuicheng Iron and Steel Co., Ltd., Guizhou, China), and others, as a system of gelling materials. The focus of this study is on the impact of vanadium-titanium slag dosing on the performance of these gelling materials, with a view to exploring the impact of gelling material properties on the hydration of the gelling material. The study aims to provide a theoretical basis and technical support for the large-scale utilisation of vanadium-titanium slag.

## 2. Results and Discussion

### 2.1. Compressive Strength Analysis

**Figure 2:**
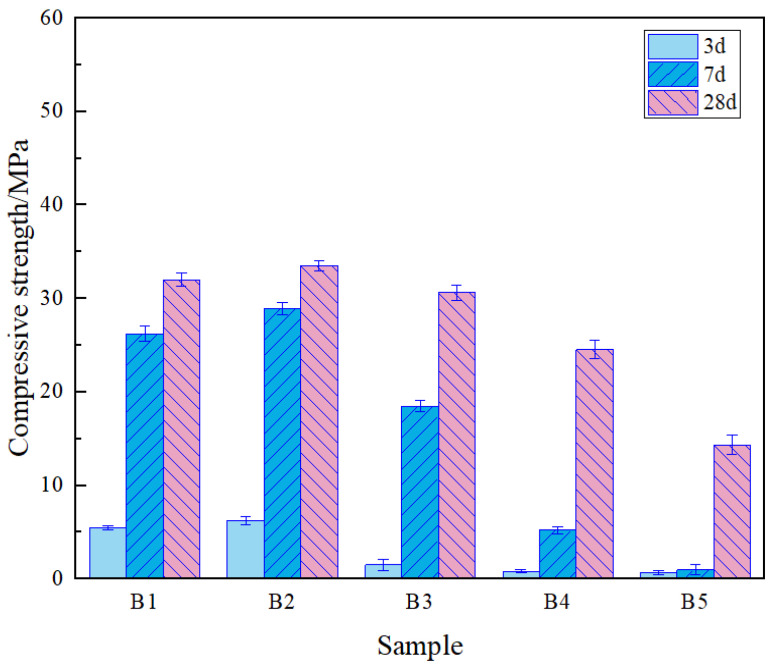
Compressive strength of vanadium-titanium slag-doped net paste specimens at all ages.

The activity test scheme and results for vanadium-titanium slag are shown in Table 3, from which the optimal grinding time was determined to be 80 min. Whereupon, experiments were conducted with vanadium-titanium slag replacing different amounts of slag, as illustrated in Table 4. As illustrated in Figure 2, the test results corresponding to the uniaxial compressive strength of each specimen at 3 d, 7 d and 28 d of curing are presented. It is evident that with the extension of hydration age from 3 to 28 days, there was an incremental increase in compressive strength of each specimen with varying vanadium-titanium slag dosage. Notably, the B2 specimen with 10.5% vanadium-titanium slag dosage exhibited a compressive strength of 33.50 MPa at 28 days, suggesting the sustained progression of the hydration reaction. In consideration of the impact of the vanadium-titanium slag factor, the dosage of the gel material exhibited a discernible effect on its compressive strength. The replacement of a proportion of slag with vanadium and titanium slag resulted in an initial enhancement in the specimen’s overall strength, which subsequently declined. When the dosage of vanadium-titanium slag is 10.5%, the strength of the specimen at all curing ages is significantly improved, and the strength performance reaches the optimum level, which is notably superior to that of the specimen prepared solely with slag (vanadium-titanium slag dosage of 0%). However, it was observed that when the proportion of vanadium-titanium slag doping exceeded 21%, there was a precipitous decline in the strength properties of the specimen. It was found that vanadium-titanium slag completely replaced the B5 specimen, resulting in a 28-day compressive strength of only 14.33 MPa. The 100% replacement group displayed the most pronounced error bars across all formulations, implying that complete dependence on vanadium-titanium slag could result in reduced uniformity in the hydration process and final mechanical performance.

The above situation demonstrates that a mixture of physically modified vanadium-titanium slag and desulfurization gypsum, in addition to a combination of slag-steel slag and desulfurization gypsum, exhibits enhanced hydration reaction properties. This enhancement is attributable to the synergistic effect of the hydration activity of the vanadium-titanium slag in the steel slag and desulfurization gypsum, which is facilitated by the joint action of these elements. The replacement of conventional commercial slag with vanadium-titanium slag in the production of low-cost, low-carbon gelling materials is a significant development. The present study provides a basis for the partial replacement of conventional commercial slag, with a view to its utilisation in the production of low-cost, low-carbon gels. A consideration of the performance of each specimen over time, specifically at 7 d and 28 d, alongside the utilisation of vanadium-titanium slag to the greatest extent possible, has led to the determination that the B2 scheme, involving a ratio of 10.5:31.5:42:16, constitutes a rational approach. This scheme encompasses the use of vanadium-titanium slag, slag, steel slag, and desulphurization gypsum.

### 2.2. Micro-Analysis

The present study examined the effect of vanadium-titanium slag dosing on the performance of the vanadium-titanium slag-slag-steel slag-desulfurization gypsum system of gel materials, with a view to determining the reasonable proportioning scheme of gel materials. Indeed, the performance manifestations of the gel materials are determined by the complex hydration reaction between the raw materials and the type and structure of the hydration products. In this section, the specimens of B1 (not doped with vanadium-titanium slag), B2 (highest strength) and B5 (lowest performance) will be studied. These three samples cover three core performance states: baseline, optimal, and worst-case, which can comprehensively reflect the effect of vanadium-titanium slag addition on the system’s performance. Sample B1, as the baseline group, is used to compare the performance changes after vanadium-titanium slag addition. Samples B2 and B5 represent the optimal and worst-case states, respectively: the former corresponds to the mix proportion with the best performance, while the latter refers to the mix proportion with the poorest performance. By comparing these two samples, the influence of vanadium-titanium slag dosage on performance can be clearly demonstrated. The compressive strength of B2 is significantly higher than that of B3; the performance contrast between B2 and B5 is even more intuitive and distinct. The hydration mechanism of the gel materials in this system will be analysed and compared using XRD, FT-IR and SEM-EDS.

#### 2.2.1. XRD Analysis

**Figure 3:**
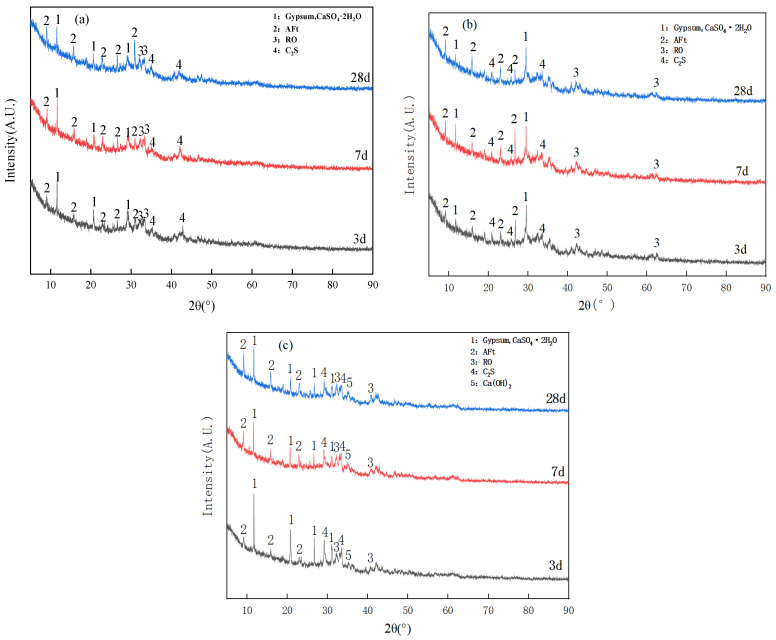
XRD patterns of each specimen at different conservation ages: (**a**) B1, (**b**) B2, (**c**) B5.

Figure 3 shows the XRD patterns of the B1, B2 and B5 specimens exhibit variation with respect to their hydration ages. In conclusion, it is evident that the substances exhibiting discernible crystal characteristic diffraction peaks in the specimen can be categorised into three distinct classifications. The first category comprises hydration products, chiefly ettringite and Ca(OH)_2_. The second category consists of unreacted substances, primarily C_2_S and dihydrate gypsum. The third category includes inert RO phases that are incapable of undergoing reaction. Furthermore, a broadened “convex packet” has been identified in the 2θ = 25~35° interval in both B1 and B2, indicating the generation of amorphous C–S–H/C–A–S–H [21].

A comparison of B1 and B2, with the extension of hydration time from 3 d to 7 d, reveals a gradual increase in the intensity of the diffraction peaks of ettringite, which stabilises at 28 d. However, the characteristic peaks of B2 specimens at 3 d and 7 d are clearly stronger than those of B1, indicating that the appropriate amount of vanadium-titanium slag doping promotes the occurrence of the hydration reaction in the early and middle stages. Furthermore, the ‘convex packing’ of the C–S–H/C–A–S–H gels in Figure 3a is more pronounced than that in Figure 3b, and these gels are able to encapsulate or fill the structural pores of the ettringite and inert phases. The combination of these two aspects of the hydration reaction and its products contributes to the superior performance of B2 over B1 in terms of strength [22].

As illustrated in Figure 3c, in contrast to B1 and B2, Ca(OH)_2_ was observed in the B5 specimens at all ages. The diffraction peak intensities of dihydrate gypsum were notably high, indicating insufficient participation in the hydration reaction, leading to the low diffraction peak intensity of calcite as a hydration product. Additionally, the “convex packet” phenomenon of the C–S–H/C–A–S–H gel was not evident. The phenomenon of “convex package” of C–S–H/C–A–S–H gel is not immediately evident. The result obtained can be attributed to the presence of excess vanadium-titanium slag, which has been shown to diminish the efficiency of slag mixing. Due to the presence of a large amount of inert and low-activity components such as titanium dioxide, perovskite, and ilmenite in vanadium-titanium slag, its activity is low. In contrast, traditional slag contains a high content of glassy phase, which is exactly the key condition for providing hydraulic activity. Consequently, the relative proportion of Ca(OH)_2_ dihydrate gypsum resulting from the excitation conditions is increased. However, it is challenging to observe this phenomenon during the full hydration reaction, which may lead to a reduction in the strength of the specimen.

#### 2.2.2. FT-IR Analysis

**Figure 4:**
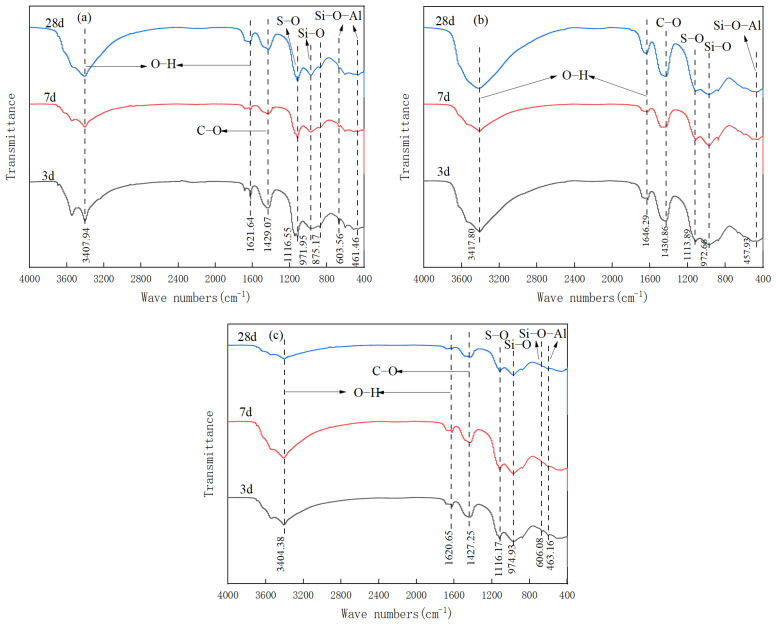
FT-IR patterns of each specimen at different conservation ages: (**a**) B1, (**b**) B2, (**c**) B5.

Figure 4 shows that the FT-IR patterns of the B1, B2 and B5 specimens vary according to their respective hydration ages. The peak near 3400 cm^−1^ appears as a broad and strong single peak without splitting, which corresponds to the stretching vibration peak of the O–H bond in the crystalline water of calcium silicate gel. The peak near 1600 cm^−1^ is relatively sharp, which is the bending vibration peak of the O–H bond in the crystalline water of ettringite [23]. Furthermore, asymmetric vibration peaks of the C–O bond of [CO_3_^2−^] have been identified at approximately 1400 cm^−1^, while S–O asymmetric vibration peaks of the [SO_4_^2−^] in desulfurization gypsum have been found at around 1100 cm^−1^. Additionally, symmetric stretching vibration peaks of the silicon–oxygen tetrahedral Si–O bond have been observed near 970 cm^−1^ and 460 cm^−1^, and asymmetric stretching vibration peaks of Si–O–Al stretching vibrational peaks and asymmetric stretching vibration peaks of Si–O bonds of silica–oxygen tetrahedra have been identified at approximately 970 cm^−1^. As demonstrated in Figure 4, the asymmetric stretching vibrational bands of Si–O–Al are observed at 600 cm^−1^ and 460 cm^−1^ [24], exhibiting a symmetrical pattern. A comparison of the shapes and positions of the main vibrational peaks of each specimen reveals that they are essentially similar, with disparities in peak intensity. Specifically, the intensity of the vibrational peaks of the O–H and Si–O bonds of B2 exceeds that of B1, the intensity of the vibrational peaks of the S–O bond is lower than that of B1, and the vibrational peaks of these bonds of B5 are not prominent. This indicates that while the hydration products of the three are essentially equivalent, the hydration rate and the number of products differ.

In general, the O–H vibrational peak transmittance decreased as the hydration time was extended from 3 d to 7 d, reaching a plateau at 28 d. This phenomenon can be attributed to the increase in the number of O–H bonds in the water of crystallisation in the hydration products, which results in the absorption of the IR waves of the corresponding wavelengths. This indicates that the hydration reaction was intense in the middle and late stages of B1 and B2, leading to the formation of hydration products such as C–S–H/C–A–S–H gels and ettringite. The number of hydration products, including C–S–H/C–A–S–H gels and ettringite, is increasing. In comparison, the peak O–H bond strength of B2 is significantly higher than that of B1, the asymmetric stretching vibrational peak of Si–O is larger than that of B1, and the vibrational peak of S–O is lower than that of B1, suggesting that gypsum dihydrate participates more fully in the hydration reaction in B2 than in B1, and generates more C–S–H/C–A–S–H gels and calixarene. This finding serves to corroborate the XRD analysis results from another perspective, thereby elucidating the intrinsic reason for the superior performance of B2 over B1.

As illustrated in Figure 4c, in contrast to B1 and B2, B5 exhibits weak O–H characteristic absorption peaks and asymmetric stretching vibrational peaks of the silica–oxygen tetrahedron Si–O, while the S–O vibrational peaks are notably high. This suggests that gypsum dihydrate does not participate in the hydration reaction adequately, resulting in the formation of limited C–S–H/C–A–S–H gels and ettringite as hydration products. Furthermore, the transmittance corresponding to the symmetric stretching vibrational bands of the Si–O–Al bond in B5 remained essentially constant at 7 d of hydration, subsequently disappearing after 28 d, suggesting that the Si–O–Al bond was broken [25]. Consequently, B5 is challenging to undergo sufficient hydration reaction, even under the excitation conditions provided by Ca(OH)_2_ and dihydrate gypsum, and the strength of the specimen is bound to decrease.

#### 2.2.3. SEM and EDS Analysis

**Figure 5:**
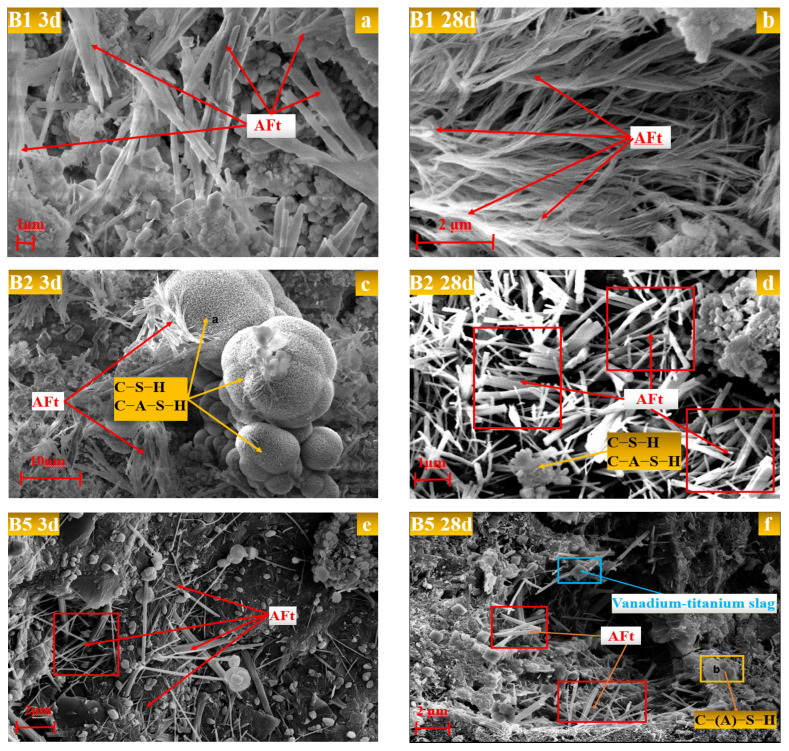
Morphology of hydration products at different ages: (**a**) B1 for three days, (**b**) B1 for twenty-eight days, (**c**) B2 for three days, (**d**) B2 for twenty-eight days, (**e**) B5 for three days, (**f**) B5 for twenty-eight days.

Figure 5 shows the SEM electron micrographs of specimens B1, B2, and B5 at different hydration ages, and Table 1 shows the results of the EDS elemental analyses at point c (Figure 5c) and point f (Figure 5f).

As demonstrated in Figure 5a,c, the hydration reaction of B1 and B2 had occurred at the age of 3 d. The short columnar ettringite in B2 was encapsulated by the clustered C–S–H/C–A–S–H gel, and some fine vanadium-titanium slag particles that did not participate in the hydration reaction were either encapsulated by the gel or filled between the ettringite crystals. These factors provided conditions for the formation of the specimen’s strength at an early stage. The configuration of the wrapping and filling structure is conducive to the development of the specimen’s initial strength. In comparison with B2, B1 exhibits a comparatively less rigid structure, resulting in diminished early strength. However, B1 does demonstrate the development of ettringite crystals and a modest amount of flocculated product C–S–H/C–A–S–H gel generation.

As demonstrated in Figure 5b,d, the generation of ettringite and C–S–H/C–A–S–H gels was observed to increase further at the age of 28 days for B1. The short columnar ettringite vanadate was found to be adhered and intertwined by a substantial number of calcium silicate (alumina) hydrate gels, resulting in the formation of a dendritic bond. This process led to the establishment of a more structurally robust cementation system and a subsequent increase in the strength of the specimens [26]. In comparison with B1, the quantity of ettringite in B2 hydrated to 28 d exhibited a substantial increase, with the crystal length attaining a length of more than 10 μm. The C–S–H/C–A–S–H gel formed a reticulated structure, characterised by the presence of short columnar ettringite in a dispersed manner. This structural feature led to the enhancement of the strength properties of B2, in comparison to B1. This observation suggests that the utilisation of a vanadium-titanium slag-slag-steel slag-desulfurization gypsum system, with the incorporation of an optimal amount of physically modified vanadium-titanium slag, results in a superior hydration reaction effect when compared to the use of a slag-steel slag-desulfurization gypsum system.

As illustrated in Figure 5e, in contrast to B1 and B2, B5 exhibits minimal hydration at 3 d, with only negligible amounts of needle-and-rod ettringite and C–S–H/C–A–S–H gels being evident. However, a substantial quantity of unhydrated raw materials, such as vanadium-titanium slag particles, is discernible. As illustrated in Figure 5f, the number of hydration products of B5 at 28 days of age remains minimal. While ettringite development is evident, its morphology is characterised by shortness and dispersion. This finding demonstrates that the multi-solid waste synergistic reaction of vanadium-titanium slag, slag, steel slag and desulfurization gypsum tetrameric system promotes the generation and growth of hydration products, ettringite, and C–S–H/C–A–S–H gel.

#### 2.2.4. Hydration Reaction Mechanism

**Figure 6:**
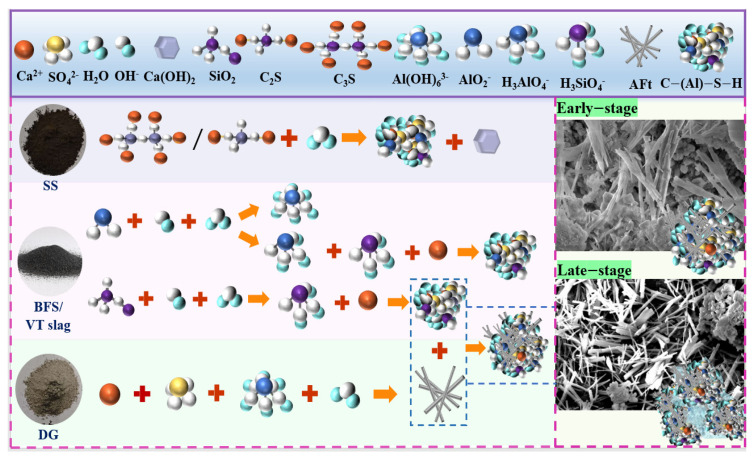
Schematic diagram of the hydration mechanism of the quaternary system.

Vanadium-titanium slag, steel slag and desulfurization gypsum constitute a system of gel materials of the above hydration products. These materials are a variety of raw materials for the hydration and depolymerisation of the basis through the hydration reaction. The number of these products and their structural relationships, in combination, determine the macroscopic properties of the gel materials. The hydration mechanism of the system is illustrated in Figure 6. The dashed squares in the figure denote that ettringite and gel interweave with each other, providing support for the strength of the system.

The hydration of C_2_S and C_3_S in steel slag has been shown to generate Ca(OH)_2_, thereby establishing an alkaline environment for the system. In addition, the process has been demonstrated to yield C–S–H/C–A–S–H gel, providing an initial strength for the system. Desulfurization gypsum has been observed to supply sufficient Ca^2+^ and SO_4_^2−^ for the system through a hydrolysis reaction. Collectively, these reactions establish an alkaline and sulfate excitation condition for the system, thus facilitating the desired chemical reactions. Slag and vanadium-titanium slag, as potentially reactive substances which are rich in silicates and aluminates, have been shown to depolymerise the Si–O and Al–O bonds in their glassy bodies by means of OH groups provided by the hydrolysis of steel slag. This process releases silicate ions (reactive SiO_2_) and aluminate ions (AlO_2_^−^), which undergo secondary bonding in the alkaline environment of the liquid phase to produce silicate–oxygen tetrahedron ions ([H_3_SiO_4_]^−^) and alumina–oxygen tetrahedron ions ([H_3_AlO_4_]^2−^). In the alkaline environment of the liquid phase, they undergo secondary bonding to produce silica–oxygen tetrahedral ions with a tetrahedral structure ([H_3_SiO_4_]^−^) and aluminium–oxygen tetrahedral ions ([H_3_AlO_4_]^2−^), as well as aluminium–oxygen octahedral ions with an octahedral structure. The presence of an octahedral structure, specifically [Al(OH)_6_]^3−^, has been identified in the liquid phase, where it combines with SO_4_^2−^ and Ca^2+^ to yield alumina ettringite (ettringite), in addition to the formation of more C–S–H and C–A–S–H gels. The fundamental process of these reactions is outlined below:

C_2_S + H_2_O→C–S–H + Ca(OH)_2_ (1)

SiO_2_ + OH^−^ + H_2_O→[H_3_SiO_4_]^−^ (2)

AlO_2_^−^ + OH^−^ + H_2_O→[H_3_AlO_4_]^2−^ (3)

AlO_2_^−^ + OH^−^ + H_2_O→[Al(OH)_6_]^3−^ (4)

6Ca^2+^ + 3SO_4_^2−^ + 2Al(OH)_6_^3−^ + 26H_2_O→3CaO∙Al_2_O_3_∙3CaSO_4_∙32H_2_O (Ettringite) (5)

[H_3_SiO_4_]^−^ + Ca^2+^→C–S–H (6)

[H_3_SiO_4_]^−^ + [H_3_AlO_4_]^2−^ + Ca^2+^→C–A–S–H (7)

## 3. Conclusions

By constructing a quaternary cementitious system consisting of vanadium-titanium slag, slag, steel slag, and desulfurization gypsum, this paper provides a scientific basis for the resource utilisation of vanadium-titanium slag. Meanwhile, the synergistic effect of the quaternary cementitious system during the hydration process is revealed, which is of great significance for promoting comprehensive resource utilisation and environmental protection. In this study, the main conclusions are as follows:

To address the issue of low reactivity of vanadium-titanium slag, mechanical force activation can enhance the reactivity of vanadium-titanium slag, thereby optimising its role in solid waste-based cementitious material systems. By comprehensively considering the activity index and energy consumption cost, it is determined that the optimal grinding time for vanadium-titanium slag is 80 min, and the most desirable performance is achieved when its specific surface area reaches 454.68 m^2^/kg.

Through experiments investigating the replacement of slag with varying dosages of vanadium-titanium slag, with the performance of specimens at 7 and 28 days as key evaluation criteria and the maximisation of vanadium-titanium slag utilisation as a core objective, the optimal mix proportion was determined. Specifically, when the mass ratio of vanadium-titanium slag:slag:steel slag:desulfurization gypsum was 10.5:31.5:42:16, and the water binder ratio was 0.32, the system achieved the best comprehensive performance, and the 28-day compressive strength of the specimen reached 33.50 MPa.

The hydration mechanism was investigated by means of microscopic analysis techniques; in the initial phase (3 d) of the hydration reaction, the system comprising vanadium-titanium slag, slag, steel slag, and desulfurization gypsum yielded hydration products; however, the hydration rate was found to be slow. Conversely, in the subsequent phases (7 d, 28 d) of the hydration reaction, there was a substantial increase in both the hydration rate and the number of hydration products. Vanadium-titanium slag is a by-product of the hydration of steel slag, desulfurization gypsum and sulfate excitation. The generation of ettringite, C–S–H/C–A–S–H gel and other major hydration products, ettringite and part of the unreacted vanadium-titanium slag occur as a result of the intertwining of calcium silicate (alumina) hydroxide gel wrapped. This process leads to the formation of dendritic stabilisation of the structure, which provides a system for the formation of strength under the given conditions.

## 4. Materials and Methods

### 4.1. Materials

**Figure 7:**
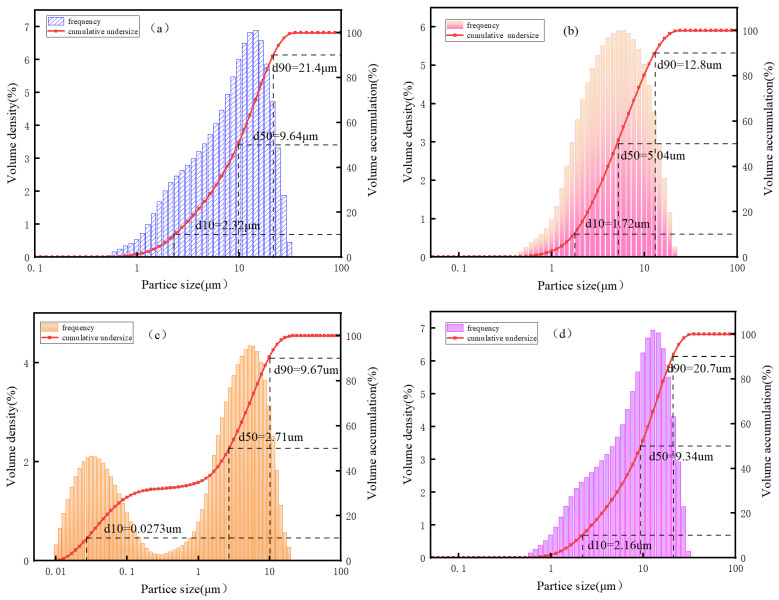
The particle size distribution of the slag: (**a**) vanadium-titanium slag, (**b**) slag, (**c**) steel slag, (**d**) desulfurization gypsum.

**Figure 8:**
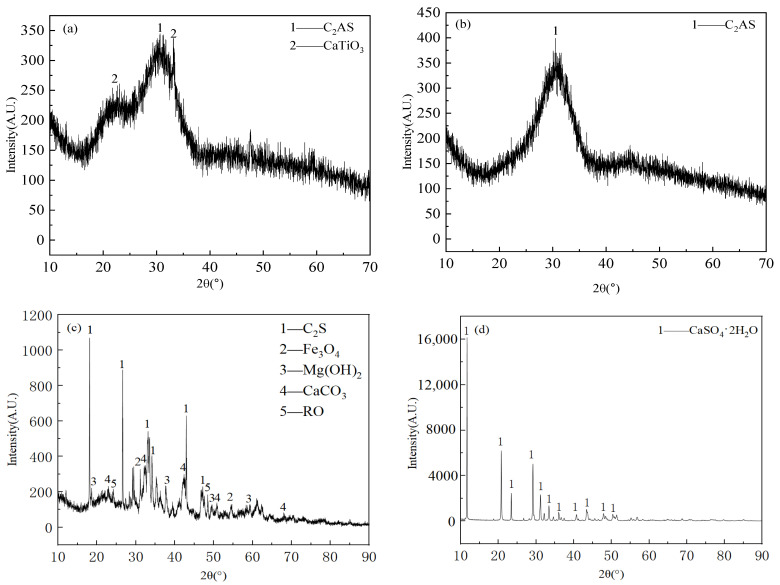
XRD patterns of raw materials: (**a**) vanadium-titanium slag, (**b**) slag, (**c**) steel slag, (**d**) desulfurization gypsum.

The Materials and Methods section presented here is provided to support the analysis in the preceding Section 2. The primary raw materials employed in this study encompass vanadium-titanium slag, slag, steel slag and desulfurization gypsum. Vanadium-titanium slag is the secondary smelting slag that is produced following the vanadium extraction process at Shougang Shuicheng Iron and Steel Co., Guizhou, China. The vanadium-titanium slag exhibited a black colouration upon completion of the drying and milling processes, with a measured density of 3.09 kg/m^3^. The results of the chemical composition analysis of the slag are shown in Table 2. The particle size distribution of the slag is shown in Figure 7. In comparison with conventional slag, the TiO_2_ content of the former was found to be elevated, reaching 15.94%, and the alkalinity coefficient Mo = W(CaO + MgO)/W(SiO_2_ + Al_2_O_3_) = 1.63 > 1, indicating its classification as an alkaline slag. Furthermore, the activity coefficient, γ = W(CaO + MgO + Al_2_O_3_)/W(SiO_2_ + MnO + TiO_2_) = 1.303 > 1.2 was determined, suggesting its inherent activity [27]. The XRD pattern of vanadium-titanium slag is shown in Figure 8a. The mineral phase of vanadium-titanium slag is comparable to that of ordinary slag, predominantly characterised by a glassy state, exhibiting a certain degree of hydration activity. This provides the potential for the occurrence of the hydration reaction, in addition to a minor presence of gehlenite and perovskite.

The blast furnace slag was obtained from Shougang Shuicheng Iron and Steel Co., Guizhou, China. It was subjected to grinding for a duration of 90 min in a small ball mill, resulting in a specific surface area of 556.03 m^2^/kg and a density of 2.97 kg/m^3^. The chemical composition analysis results of the slag are shown in Table 2, along with its alkalinity coefficient. It can be demonstrated that Mo = 1.197 > 1, thus classifying the substance as an alkaline slag. Furthermore, the activity coefficient, K, of the slag is found to be greater than 1.2, thereby indicating that the slag is deemed to be of a high activity classification. The XRD analysis of the slag is shown in Figure 8b, which demonstrates that, with the exception of a weak gehlenite crystalline peak, there is no obvious crystalline phase.

The steel slag under consideration is the converter slag of Shougang Shuicheng Iron and Steel Co., Guizhou, China. Following a period of 80 min of grinding in a small ball mill, the specific surface area was determined to be 498.49 m^2^/kg, and the density was found to be 3.51 kg/m^3^. The chemical composition of the slag is analysed in Table 2, and its alkalinity coefficient, Mo = 2.987 > 1, is determined to be greater than 1, thus classifying it as an alkaline steel slag. The XRD analysis results are displayed in Figure 8c, and the physical phases are primarily C_2_S and C_3_S, in addition to mineral phases such as magnetite, magnesite, calcite and RO phase. Among these phases, C_2_S and C_3_S have potential hydration activity, while generating Ca(OH)_2_, providing an alkaline environment for the system. This can provide an alkaline environment for the system. Desulfurization gypsum is sourced from Shougang Shuicheng Iron and Steel Co., Guizhou, China. Following a 30 min grinding process in a small ball mill, the specific surface area was determined to be 473.19 m^2^/kg, while the density was found to be 2.36 kg/m^3^. The results of the chemical composition analysis of the desulfurization gypsum are shown in Table 2, and its main components are CaO and SO_3_. The results of the XRD analysis are shown in Figure 8d, and the main mineral phase is dihydrate gypsum (CaSO_4_·2H_2_O).

### 4.2. Activation of Vanadium-Titanium Slag

As previously stated, vanadium-titanium slag exhibits a reduced level of activity in comparison to conventional slag. In order to enhance the activity of vanadium-titanium slag and its application effect in gelling materials, the research in this paper is based on the following two approaches: Firstly, the vanadium-titanium slag was subjected to mechanical activation by grinding in order to enhance its activity. Secondly, through the hydration reaction with steel slag, desulfurization gypsum and other materials in the gelling material system, its gelling activity is further stimulated. It is imperative to note that the primary objective of this study is to conduct experimental investigations into the grinding process of vanadium-titanium slag. The central aim of this undertaking is to ascertain the most reasonable specific surface area and grinding time for this particular material.

### 4.3. Methods

The experimental programme of grinding and mastic sand, as illustrated in Table 3, was designed in accordance with the provisions of GB/T 17671-2021, entitled “Cement Mastic Sand Strength Test Methods” [28]. The objective of this programme was to prepare mastic sand specimens and to ascertain their uniaxial compressive strength at 3 d, 7 d, and 28 d, in addition to calculating the corresponding activity index. In this experiment, specimen S0 is a control specimen prepared from a benchmark cement (P.O. 42.5 ordinary silicate cement) for comparison with the vanadium-titanium slag-based specimen. Specimens S1–S4 consist of samples prepared by substituting 50% of the cement with vanadium-titanium slag of different grinding durations. To ensure the reliability of the test results and the strength of the specimen, each specimen was repeated three times, and the average value of its compressive strength was taken. The sand used for the experiment was China ISO standard sand.

The results of the specific surface area determination of vanadium-titanium slag milling at different times are shown in Table 3. It has been demonstrated that in order to achieve a specific surface area in excess of 450 m^2^/kg, a grinding time in excess of 80 min is a prerequisite. This finding serves to compound the conclusion that the substance in question is characterised by inadequate grindability. Furthermore, an examination of the data reveals a tendency for the specific surface area to initially increase rapidly, followed by a subsequent decline. This observation indicates that an excessively protracted grinding time may not necessarily result in a more efficacious grinding outcome. It is therefore imperative that a judicious grinding time is employed, taking into consideration the associated grinding costs.

**Figure 9:**
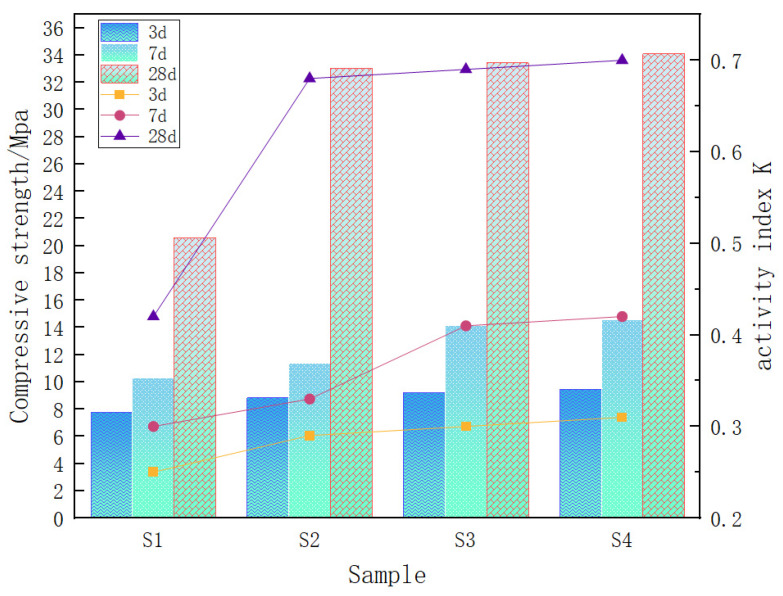
Comparison of compressive strength and activity index of vanadium-titanium slag grit samples at different ages.

Figure 9 shows the comparison of compressive strength and activity index at different ages from S1 to S4. The activity index K is calculated as the ratio of the compressive strength of mortar specimens incorporating the test material to that of reference cement mortar specimens, multiplied by 100%. It is evident that the activity indices of the specimens at three days of maintenance are generally low and exhibited minimal variation, suggesting that the milling treatment did not significantly enhance the activity index of vanadium-titanium slag at three days. However, as the maintenance age increased, the activity index increased for all specimens. Furthermore, the activity indices of S2~S4 at 28 days remained relatively constant, while the activity increased substantially. Additionally, the activity indices and the increase in S2~S4 at 28 days were considerably higher than those of S1, indicating that increasing the specific surface area can enhance the activity of vanadium-titanium slag in the middle and late stages.

As illustrated in Figure 9, the compressive strength indicators of the vanadium-titanium slag activity-specific embodiment also demonstrate a gradual increase in performance with the growth of age, as evidenced by the increasing index values of S1 to S4. Furthermore, the increase in compressive strength is more pronounced in the middle and late stages compared to the early stages. This phenomenon can be attributed to the presence of vanadium-titanium slag. In addition to their own hydration activity, mechanical force activation leads to the formation of slag particles due to lattice distortion and amorphous transformation. The resulting amorphous SiO_2_ and Al_2_O_3_ components are then dissolved and involved in the subsequent hydration reaction process, through the generation of the corresponding hydration products. This process contributes to the specimen’s varied range of strengths.

To summarise, the milling of vanadium-titanium slag has been demonstrated to enhance its activity index, thereby optimising the performance of the resultant gel materials. Taking into consideration the activity index, grinding energy and cost, it is evident that when the specific surface area of vanadium-titanium slag is 454.68 m^2^/kg. The specific surface area of 454.68 m^2^/kg is considered reasonable for physically stimulating the hydration activity of vanadium and titanium slag when the grinding time is 80 min. At this time, the 28-day activity index of vanadium and titanium slag reached 68%, which is already close to the index value of S75 slag powder [29].

The experimental study of vanadium-titanium slag milling revealed a milling effect of 68% activity index under the condition of an 80 min milling time. It is imperative that further exploration be undertaken in order to ascertain whether this modified vanadium-titanium slag is capable of exerting its hydration activity under the excitation of steel slag and desulfurization gypsum, in a manner analogous to that of ordinary slag. For this reason, the present study proposes the introduction of a ternary system comprising vanadium and titanium slag, slag, steel slag and desulfurization gypsum into the slag-steel slag-desulfurization gypsum system. The objective of this study is to explore the alkali excitation in steel slag, desulfurization gypsum, and the combined excitation of sulfate, vanadium-titanium slag in the gelling material system of the hydration activity. The central focus of this study is on the exploration of vanadium-titanium slag mixing (partially replacing the ordinary slag) on the hydration of the solid waste-based gelling materials and the performance of the impact of solid waste-based gelling materials. The overarching aim of this study is to provide a basis for the development and utilisation of vanadium-titanium slag-slag-steel slag-desulfurization gypsum system gel materials and vanadium-titanium slag resource utilisation. In accordance with the preceding research outcomes of this collective on the ternary system of slag, steel slag and desulfurization gypsum, a series of fundamental proportioning schemes was initially established. Specifically, the proportion of slag to steel slag to desulfurization gypsum was determined to be 42:42:16. Subsequently, the slag was partially or completely substituted with vanadium-titanium slag, thereby facilitating the formulation of an experimental scheme for the quaternary system, as illustrated in Table 4.

In accordance with the provisions stipulated in GB/T 17671-2021, “Gels Sand Strength Test Methods”, the experimental programme was meticulously designed to prepare net mortar specimens at specific time points (3 d, 7 d, 28 d) to assess the uniaxial compressive strength. The index was utilised as the basis for evaluation. The primary objective was to investigate the impact of vanadium-titanium slag mixing (the proportion of replacement of slag) on the performance of solid waste-based gel materials and the hydration process. The secondary objective was to ascertain the optimal amount of vanadium-titanium slag replacement in slag mixing. The optimum dosage of vanadium-titanium slag as an alternative to slag is then determined.

## Figures and Tables

**Table 1:** Elemental composition of points c and f in Figure 5 (Wt. %).

Position	Content of Individual Elements						
	O	Mg	Al	Si	S	Ca	Ti
a	38.26	5.02	6.70	12.14	0.00	34.74	0.06
b	39.08	0.00	10.68	26.15	0.00	20.89	4.09

**Table 2:** The chemical composition of materials (Wt. %).

	CaO	SiO_2_	Al_2_O_3_	MgO	TiO_2_	Fe_2_O_3_	MnO	V_2_O_5_	SO_3_	Cl
Vanadium-titanium	33.78	25.23	11.64	9.11	15.94	1.60	0.69	ND	0.92	0.02
Blast Furnace Slag	41.37	29.87	11.85	8.56	1.75	2.50	0.43	ND	1.31	0.03
Steel slag	41.74	13.89	3.36	9.78	2.29	19.51	3.78	1.12	0.59	0.05
Desulfurization gypsum	49.30	1.53	0.23	1.08	0.04	0.41	0.01	ND	45.58	0.43

**Table 3:** Experimental plan for the activity of vanadium-titanium slag.

Number	Grinding Time/min	Specific Surface Area/m^2^/kg	Cement/g	V-T Slag/g	S/g	Water/mL
S0	—	—	450	—	1350	225
S1	60	403.17	225	225	1350	225
S2	80	454.68	225	225	1350	225
S3	100	494.64	225	225	1350	225
S4	120	541.67	225	225	1350	225

**Table 4:** Experimental programme of vanadium-titanium slag-doped net paste.

Number	Composition of Gels Materials (wt. %)				Quality (g)	W/b Ratio	Water	Total Mass (g)
	V-T Slag	Slag	SS	Desulfurization Gypsum				
B1	0	42	42	16	758	0.32	242	1000
B2	10.5	31.5	42	16	758	0.32	242	1000
B3	21	21	42	16	758	0.32	242	1000
B4	31.5	10.5	42	16	758	0.32	242	1000
B5	42	0	42	16	758	0.32	242	1000

## Data Availability

The original contributions presented in this study are included in the article. Further inquiries can be directed to the corresponding authors.
